# Safety, pharmacokinetics, and pharmacodynamics of SHR7280, an oral gonadotropin-releasing hormone receptor antagonist, in healthy men: a randomized, double-blind, placebo-controlled phase 1 study

**DOI:** 10.1186/s12916-023-02834-6

**Published:** 2023-04-03

**Authors:** Xin Li, Feifei Sun, Xiaolei Zhang, Pingping Lin, Kai Shen, Yu Shen, Lingyu Ma, Yu Cao, Chenjing Wang

**Affiliations:** 1 Phase I Clinical Research Center, The Affiliated Hospital of Qingdao University, 16 Jiangsu Road, Qingdao, 266003 China; 2 Jiangsu Hengrui Pharmaceuticals Co., Ltd, Shanghai, China

**Keywords:** Gonadotropin-releasing hormone antagonists, SHR7280, Trial in healthy men, Safety, Pharmacokinetics, Pharmacodynamics

## Abstract

**Background:**

Gonadotropin-releasing hormone (GnRH) antagonists are a promising therapeutic approach for treating hormone-dependent prostate cancer. Currently, the mainstream GnRH antagonists are polypeptide agents administered through subcutaneous injection. In this study, we evaluated the safety, pharmacokinetics (PK), and pharmacodynamics (PD) of SHR7280, an oral small molecule GnRH antagonist, in healthy men.

**Methods:**

This phase 1 trial was a randomized, double-blind, placebo-controlled, and dose-ascending study. Eligible healthy men were randomized in a 4:1 ratio to receive either oral SHR7280 tablets or placebo twice daily (BID) for 14 consecutive days. The SHR7280 dose was initiated at 100 mg BID and then sequentially increased to 200, 350, 500, 600, 800, and 1000 mg BID. Safety, PK, and PD parameters were assessed.

**Results:**

A total of 70 subjects were enrolled and received the assigned drug, including 56 with SHR7280 and 14 with placebo. SHR7280 was well-tolerated. The incidence of adverse events (AEs, 76.8% vs 85.7%) and treatment-related AEs (75.0% vs 85.7%), as well as the severity of AEs (moderate AEs, 1.8% vs 7.1%) were similar between the SHR7280 group and placebo group. SHR7280 was rapidly absorbed in a dose-dependent manner, with a median *T*_max_ of each dose group ranging from 0.8 to 1.0 h on day 14 and a mean *t*_1/2_ ranging from 2.8 to 3.4 h. The PD results demonstrated that SHR7280 exhibited a rapid and dose-proportional suppression of hormones, including LH, FSH, and testosterone, with maximum suppression achieved at doses of 800 and 1000 mg BID.

**Conclusions:**

SHR7280 showed an acceptable safety profile, as well as favorable PK and PD profiles within a dose range of 100 to 1000 mg BID. This study proposes a rationale for further investigation of SHR7280 as a potential androgen deprivation therapy.

**Trial registration:**

Clinical trials.gov NCT04554043; registered September 18, 2020.

**Supplementary Information:**

The online version contains supplementary material available at 10.1186/s12916-023-02834-6.

## Background

Globally, prostate cancer represents a significant health burden for the male population, being the second most commonly diagnosed cancer and the fifth leading cause of cancer-related mortality among men in 2020 [1]. The foundational therapeutic approach for advanced prostate cancer is androgen deprivation therapy (ADT) [2–5].

In the hypothalamic–pituitary axis, the hypothalamus secretes gonadotropin-releasing hormone (GnRH), which stimulates the release of pituitary luteinizing hormone (LH) and follicle-stimulating hormone (FSH). LH, in turn, stimulates testosterone production [6]. Consequently, targeting GnRH, which is the upstream regulatory factor of testosterone, has become a feasible therapeutic strategy for prostate cancer. In the early years, GnRH agonists were the standard of care for advanced prostate cancer. However, their use was limited due to clinical flare-up of symptoms, side effects, and a delayed onset of testosterone suppression for several weeks [7–12].

An alternative option of ADT is the suppression of sex hormones by GnRH antagonists [13, 14]. GnRH antagonists compete with GnRH receptor signaling, blocking the release of LH and FSH from the pituitary and leading to a decline in testosterone production. Compared to GnRH agonists, GnRH antagonists have a rapid efficacy in testosterone castration, and the suppressed testosterone level can be quickly restored after treatment cessation [13]. Abarelix and degarelix were two pioneering GnRH antagonists, but their development and clinical use were hindered by allergic reactions and injection administration, respectively [15, 16]. Currently, the most widely used oral GnRH antagonist for advanced prostate cancer is relugolix, which received FDA approval in 2020 [17, 18]. Nevertheless, it still has some disadvantages, such as causing hot flashes, prolonging the QT interval, having embryo-fetal toxicity, and being incompatible with co-administrated of P-glycoprotein (P-gp) inhibitors and combined P-gp and strong cytochrome P450 3A (CYP3A) inducers. Thus, the development of alternative GnRH antagonists with promising efficacy and acceptable safety is warranted.

SHR7280 is a novel oral small molecular GnRH antagonist that selectively blocks the binding between endogenous GnRH and its receptor, inhibiting the synthesis and release of gonadotropins like LH and FSH, and reducing the levels of testosterone and E_2_. SHR7280 has been shown to have a short half-life in in vitro studies, ranging from 1.0 to 2.2 h in rats and from 3.2 to 4.5 h in dogs. The drug demonstrated comparable exposures between males and females. Additionally, it has good transmembrane ability and is equivalent to medium to high level of human intestinal absorption (50–70%). In animal models, SHR7280 has demonstrated fewer side effects on the cardiovascular, respiratory, and nervous systems and no genotoxicity (unpublished data). It is currently being developed for the treatment of sex hormone-dependent diseases, such as prostate cancer, endometriosis, and hysteromyoma, as well as for the prevention of premature LH surges in assisted reproduction technology procedures. Therefore, we conducted this phase 1 study to assess the safety, pharmacokinetics (PK), and pharmacodynamics (PD) of SHR7280 in healthy men.

## Methods

### Study population

Healthy men between the ages of 18 and 65 with a body mass index ranging from 18 to 30 kg/m^2^ were eligible for this study.

Major exclusion criteria included testosterone less than 12 nmol/L, a history of using GnRH agonists or GnRH antagonists within 6 months prior to screening, and a history of using any androgen or anti-androgen drugs within 5 half-life periods prior to screening. Other exclusion criteria included levels of glutamic pyruvic transaminase, glutamic oxaloacetic transaminase, or total bilirubin exceeding the upper limit of normal value at screening; chronic or serious diseases that affect drug absorption, distribution, metabolism, and excretion; positivity for hepatitis B virus, hepatitis C virus, human immunodeficiency virus antibody, or *Treponema pallidum*; positive results in nicotine or alcohol breath test, or drug screen test before administration; a history of using drugs to inhibit or induce liver drug metabolism within 1 month before administration; and consumption of caffeine, food or drink rich in purine, tobacco, grapefruit or grapefruit juice, and alcohol within 48 h before administration.

### Study design and treatment

This study was a randomized, double-blinded, placebo-controlled, dose-ascending, phase 1 trial of SHR7280 in healthy male subjects (NCT04554043).

Eligible male subjects received oral SHR7280 tablets or placebo twice daily for 14 consecutive days. Seven dose cohorts (100, 200, 350, 500, 600, 800, and 1000 mg BID) were preplanned. The starting dose, frequency of dosing, and maximum dose were determined based on the PK data and tolerability findings from a phase 1 trial of SHR7280 in healthy premenopausal women, which showed acceptable tolerability of SHR7280 within the dose range of 50 to 400 mg (registered at http://www.chictr.org.cn/, CTR20181472; unpublished data). Ten eligible subjects were enrolled in each dose cohort and randomized in a 4:1 ratio to receive either SHR7280 (*n* = 8) or placebo (*n* = 2). The randomization was performed using the Randomization and Trial Supply Management system (RTSM system; Bioknow, Beijing, China). After all subjects in the preceding lower-dose cohort completed 14 days of treatment and 2 days of observation period (on D16) or 2 days after the last dose, the Safety Review Committee (SRC) evaluated the safety data and decided on initiation of dose escalation.

Dose escalation would be terminated if any of the following criteria were met: occurrence of a serious adverse event (AE) related to SHR7280; occurrence of severe AE of the same organ system related to SHR7280 occurred in ≥ 2 subjects in the same dose group; or ≥ 50% of subjects in the same dose group having moderate or severe AEs related to SHR7280.

### Safety assessment

AEs were assessed from the initiation of drug administration to D42 ± 2 days or 28 ± 2 days after the last administration. AEs were classified according to Medical Dictionary for Regulatory Activities (MedDRA) v24.0. The severities of AEs were graded according to the National Cancer Institute's Common Terminology Criteria for Adverse Events (CTCAE) v5.0, with grade 1 being mild, grade 2 being moderate, and grades 3–5 being severe. The analyzed PK parameters included the area under the plasma concentration–time profile (AUC_0-12_), the time to maximum plasma concentration (*T*_max_), and the maximum plasma concentration (*C*_max_) on day 1, as well as half-life (*t*_1/2_), AUC, apparent volume of distribution (*V*_z_/*F*), apparent total clearance (CL/F), *C*_max_, *T*_max_, trough plasma concentration (*C*_trough_), and accumulation ratio (*R*_acc_).

### PD analysis

Blood samples for PD analysis were collected at predose (0), 2, 4, 6, 8, 10, 12, and 16 h on day 1, predose on day 2, day 3, day 7, day 11, and day 13, and predose, 2, 4, 6, 8, 10, 12, 16, 24, 36, and 48 h on day 14. The testosterone levels were detected using the chemiluminescence method with the Sciex Triple Quad 6500 + by Frontage Laboratories Co. Ltd. (Shanghai, China). The lower limit of quantification (LLOQ) for testosterone was 0.05 ng/mL. The levels of FSH and LH were measured using the Architect iSR2000 Immunoassay analyzer (the LLOQ for FSH and LH was 0.75 and 0.11 mIU/mL, respectively) and Alinity analyzers (the LLOQ for FSH and LH was 0.05 and 0.09 mIU/mL, respectively; Abbott Laboratories, Abbott Park, IL, USA) by KingMed Diagnostics Group Co., Ltd. (Guangzhou, China).

### Statistical analysis

The sample size was determined according to the “Guidelines for clinical pharmacokinetic studies of chemical drugs” by the China National Medical Products Administration, which recommends a sample size of 8 to 12 cases in each dose group for multiple-dose studies in healthy subjects. No formal statistical calculation was used to predefine the sample size.

Safety was assessed in all subjects who received at least one dose of SHR7280 or placebo. Plasma drug concentration and PK parameters were analyzed in subjects who received at least one dose of SHR7280 or placebo and had at least one qualified blood sample for plasma drug concentration and PK parameter assessment. PD parameters were evaluated in subjects who received at least one dose of SHR7280 or placebo and had at least one qualified sample for PD assessment.

Baseline characteristics, safety data, and parts of PK and PD parameters were summarized using descriptive statistics. The relationship between PK parameters and dose after log transformation was analyzed using ANOVA model. The AUC of PK and PD parameters were analyzed using a non-compartment model. Statistical analyses were conducted using SAS v9.4 and Phoenix v8.2 or higher (for PK and PD data; Certara, Princeton, NJ, USA).

## Results

### Participants

Between September 14, 2020, and May 9, 2021, a total of 321 healthy men were screened, and 70 eligible subjects were enrolled. Of these, 56 subjects were randomly assigned to receive SHR7280 (eight subjects for each dose), and 14 were assigned to receive placebo. Two subjects in the SHR7280 group (one in the 500 mg group and one in the 1000 mg group) discontinued the treatment due to subject decision. The remaining 68 subjects completed the assigned study treatment. Baseline characteristics were well-balanced across different doses of SHR7280 and the placebo group (Table 1).

**Table 1:** Baseline characteristics

	**100 mg BID (*n* = 8)**	**200 mg BID (*n* = 8)**	**350 mg BID (*n* = 8)**	**500 mg BID (*n* = 8)**	**600 mg BID (*n* = 8)**	**800 mg BID (*n* = 8)**	**1000 mg BID (*n* = 8)**	**Placebo (*n* = 14)**	**Total (*n* = 70)**
Age, years	30.1 (7.4)	30.5 (5.3)	32.9 (7.7)	29.5 (7.8)	28.5 (3.8)	31.9 (9.0)	31.1 (6.5)	28.5 (7.4)	30.2 (6.9)
BMI, kg/m^2^	23.6 (2.0)	25.7 (3.2)	23.5 (3.6)	24.1 (3.9)	22.7 (2.7)	25.0 (2.8)	22.3 (2.0)	22.9 (3.7)	23.7 (3.2)
Height, cm	170.7 (8.2)	175.8 (6.3)	174.6 (8.0)	173.3 (5.9)	170.8 (4.5)	172.2 (6.5)	169.1 (3.7)	173.1 (5.4)	172.5 (6.2)
Weight, kg	68.9 (9.0)	80.0(14.3)	71.5 (10.8)	72.3 (11.6)	66.3 (9.5)	74.2 (9.7)	63.9 (7.2)	69.0 (12.9)	70.6 (11.4)

Data are mean (SD)

### Safety

All 70 subjects were included in the safety assessment. Of these, 55 (78.6%) subjects experienced at least one AE, including 43 (76.8%) of the 56 subjects in the SHR7280 group and 12 (85.7%) of the 14 subjects in the placebo group (Table 2). The incidence of AEs did not increase with higher doses of the drug. The most frequently reported AEs in patients who received SHR7280 were increased blood bilirubin (30.4%) and increased alanine aminotransferase (16.1%). In the 100, 200, 350, 500, 600, 800, and 1000 mg BID (*n* = 8) groups of SHR7280 and the placebo group, the incidence of increased blood bilirubin was 12.5, 37.5, 37.5, 50.0, 12.5, 37.5, 25.0, and 28.6%, respectively, and the incidence of increased alanine aminotransferase was 25.0, 0, 0, 25.0, 12.5, 25.0, 25.0, and 14.3%, respectively.

**Table 2:** Safety results

	**100 mg BID (*n* = 8)**	**200 mg BID (*n* = 8)**	**350 mg BID (*n* = 8)**	**500 mg BID (*n* = 8)**	**600 mg BID (*n* = 8)**	**800 mg BID (*n* = 8)**	**1000 mg BID (*n* = 8)**	**SHR7280 (*n* = 56)**	**Placebo (*n* = 14)**
Any	8 (100)	6 (75.0)	6 (75.0)	5 (62.5)	5 (62.5)	6 (75.0)	7 (87.5)	43 (76.8)	12 (85.7)
Blood bilirubin increased	1 (12.5)	3 (37.5)	3 (37.5)	4 (50.0)	1 (12.5)	3 (37.5)	2 (25.0)	17 (30.4)	4 (28.6)
Alanine aminotransferase increased	2 (25.0)	0	0	2 (25.0)	1 (12.5)	2 (25.0)	2 (25.0)	9 (16.1)	2 (14.3)
Aspartate aminotransferase increased	1 (12.5)	0	0	2 (25.0)	1 (12.5)	1 (12.5)	2 (25.0)	7 (12.5)	1 (7.1)
Blood calcium decreased	5 (62.5)	0	0	1 (12.5)	0	0	0	6 (10.7)	0
Blood thyroid-stimulating hormone increased	0	2 (25.0)	1 (12.5)	0	1 (12.5)	1 (12.5)	0	5 (8.9)	4 (28.6)
Blood testosterone increased	0	0	0	1 (12.5)	1 (12.5)	1 (12.5)	2 (25.0)	5 (8.9)	0
Fibrin D dimer increased	1 (12.5)	0	0	0	0	2 (25.0)	0	3 (5.4)	0
Thyroxine free increased	0	0	0	0	0	2 (25.0)	1 (12.5)	3 (5.4)	0
Neutrophil count decreased	0	0	0	0	1 (12.5)	1 (12.5)	1 (12.5)	3 (5.4)	0
Diarrhea	0	1 (12.5)	0	0	0	0	2 (25.0)	3 (5.4)	0
Upper respiratory tract infection	0	0	0	1 (12.5)	0	0	2 (25.0)	3 (5.4)	0
White blood cells urine positive	0	0	0	0	1 (12.5)	1 (12.5)	0	2 (3.6)	1 (7.1)
Blood thyroid-stimulating hormone decreased	0	0	1 (12.5)	0	0	1 (12.5)	0	2 (3.6)	1 (7.1)
Thyroxine free decreased	2 (25.0)	0	0	0	0	0	0	2 (3.6)	1 (7.1)
Blood uric acid increased	0	0	1 (12.5)	0	0	0	1 (12.5)	2 (3.6)	0
Dry mouth	0	0	2 (25.0)	0	0	0	0	2 (3.6)	0
Supraventricular extrasystoles	0	1 (12.5)	0	1 (12.5)	0	0	0	2 (3.6)	1 (7.1)
Ventricular extrasystoles	0	1 (12.5)	0	1 (12.5)	0	0	0	2 (3.6)	0
Sinus tachycardia	0	0	2 (25.0)	0	0	0	0	2 (3.6)	0
Tri-iodothyronine free increased	0	1 (12.5)	0	0	0	0	0	1 (1.8)	2 (14.3)

Data are *n* (%). AEs occurred in two or more subjects in any treatment group are listed

The majority of AEs were mild in severity, and none were reported as severe. Only two moderate AEs were reported, which were both judged as treatment-related. One moderate AE was anemia from the 350 mg BID group of SHR7280, and the other was hypercholesterolemia from the placebo group, both of which resolved with appropriate intervention. No serious AEs occurred, and no AEs resulted in treatment discontinuation or death. The incidence and severity of AEs were not dose-dependent.

Forty-two (75.0%) subjects in the SHR7280 group and 12 (85.7%) subjects in the placebo group experienced treatment-related AEs, with increased blood bilirubin (30.4%) and increased alanine aminotransferase (16.1%) being the most common ones in the SHR7280 group.

### PK

**Fig. 1:**
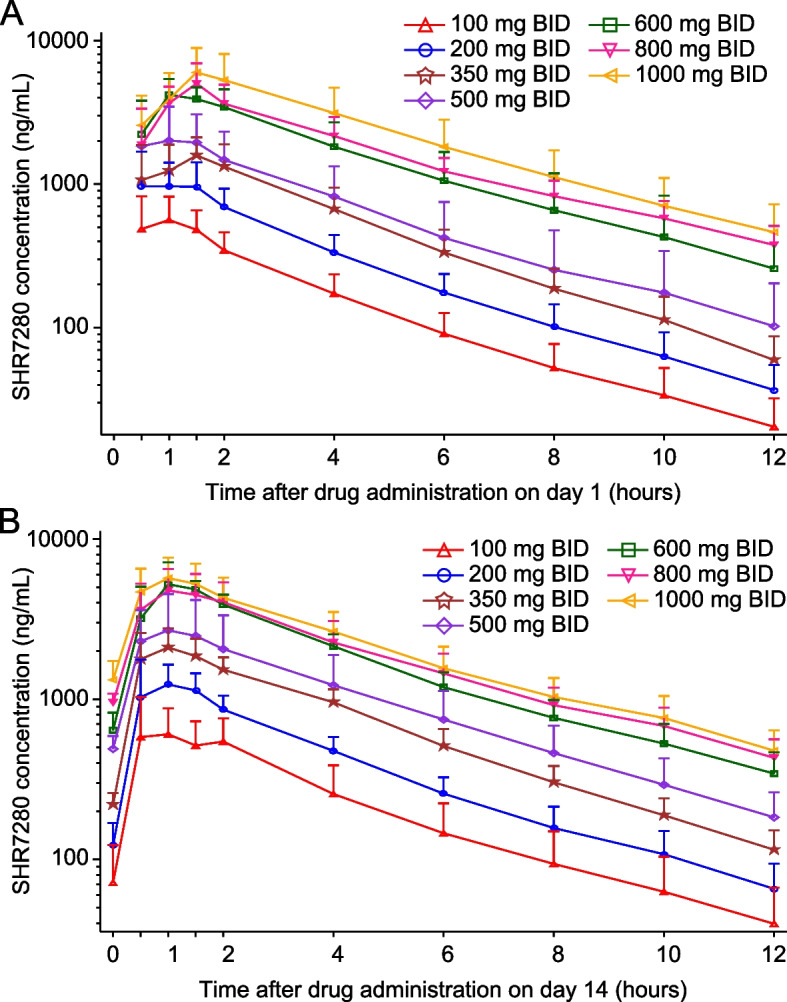
Plasma concentration–time profiles of SHR7280 on day 1 (**A**) and day 14 (**B**). Data are presented as mean (SD)

All 56 subjects who received SHR7280 administration were included in the PK analysis. The plasma concentration–time profiles of SHR7280 by dose on day 1 and day 14 are presented in Fig. 1, and PK parameters by dose are summarized in Table 3.

**Table 3:** SHR7280 PK parameters

		**100 mg BID (*n* = 8)**	**200 mg BID (*n* = 8)**	**350 mg BID (*n* = 8)**	**500 mg BID (*n* = 8)**	**600 mg BID (*n* = 8)**	**800 mg BID (*n* = 8)**	**1000 mg BID (*n* = 8)**
Day 1								
*T*_max_, h	Median (range)	0.8 (0.5–1.5)	1.0 (0.5–1.5)	1.3 (0.5–2.0)	1.0 (0.5–1.5)	1.0 (0.5–2.0)	1.5 (0.5–1.5)	1.3 (0.5–2.0)
*C*_max_, ng/mL	Mean (SD)	696 (238)	1340 (592)	1970 (267)	2600 (1760)	4500 (1160)	5150 (1840)	6300 (2690)
	GeoMean (%CV)	655 (34.1)	1200 (44.2)	1950 (13.6)	1700 (67.7)	4370 (25.8)	4860 (35.8)	5800 (42.7)
AUC_0-12 h_, h*ng/mL	Mean (SD)	1880 (658)	3610 (1100)	6110 (1390)	8030 (4830)	17,200 (6910)	19,500 (5060)	26,400 (11,100)
	GeoMean (%CV)	1790 (35.1)	3420 (30.4)	5960 (22.7)	5530 (60.1)	16,300 (40.1)	18,800 (26.0)	23,800 (42.0)
Day 14								
*T*_max_, h	Median (range)	0.8 (0.5–2.0)	1.0 (0.5–2.1)	1.0 (0.5–4.0)	1.0 (0.5–4.0)	1.0 (0.5–1.5)	1.0 (0.5–2.0)	1.0 (0.5–1.0)
*C*_max_, ng/mL	Mean (SD)	809 (255)	1430 (431)	2310 (597)	2870 (1770)	5870 (944)	5240 (1630)	6000 (1780)
	GeoMean (%CV)	776 (31.6)	1370 (30.2)	2230 (25.8)	2280 (61.9)	5800 (16.1)	4940 (31.1)	5670 (29.7)
AUC_0-12 h_, h*ng/mL	Mean (SD)	2650 (1160)	4800 (1200)	8720 (1330)	11,900 (6550)	20,900 (2900)	22,400 (6610)	25,700 (8060)
	GeoMean (%CV)	2470 (43.7)	4680 (24.9)	8630 (15.2)	10,100 (55.1)	20,700 (13.9)	21,200 (29.5)	24,300 (31.4)
*t*_1/2_, h	Mean (SD)	3.1 (0.4)	2.9 (0.5)	2.8 (0.4)	3.1 (0.4)	3.2 (0.6)	3.4 (0.3)	3.1 (0.2)
	GeoMean (%CV)	3.07 (14.3)	2.86 (18.1)	2.74 (15.3)	3.11 (13.4)	3.14 (17.7)	3.38 (8.6)	3.09 (8.0)
*V*_z_/*F*, L	Mean (SD)	189 (62.9)	179 (31.4)	164 (35.0)	287 (223)	134 (28.9)	200 (102)	197 (88.2)
	GeoMean (%CV)	179 (33.2)	176 (17.6)	160 (21.4)	222 (77.7)	131 (21.6)	184 (50.8)	184 (44.8)
*CL*/*F*, L/h	Mean (SD)	42.7 (13.9)	43.8 (9.8)	40.9 (6.1)	59.7 (39.3)	29.2 (3.7)	40.8 (20.6)	44.3 (21.0)
	GeoMean (%CV)	40.4 (32.5)	42.8 (22.4)	40.5 (14.9)	49.5 (65.9)	29.0 (12.7)	37.7 (50.6)	41.1 (47.6)
*C*_trough_, ng/mL	Mean (SD)	39.7 (27.1)	65.6 (28.8)	115 (37.2)	233 (81.0)	343 (123)	431 (131)	477 (161)
	GeoMean (%CV)	33.6 (68.4)	60.6 (43.9)	109 (32.4)	219 (34.8)	326 (35.7)	406 (30.3)	449 (33.7)
*R*_acc_	Mean (SD)	1.4 (0.3)	1.4 (0.5)	1.5 (0.6)	2.7 (2.5)	1.3 (0.4)	1.3 (0.6)	1.1 (0.4)
	GeoMean (%CV)	1.38 (18.9)	1.37 (33.4)	1.45 (37.5)	2.02 (95.1)	1.27 (29.2)	1.13 (45.8)	1.07 (36.5)

*AUC*_*0-12 h*_ Area under the concentration time curve from zero to 12 h, *CL/F* Apparent clearance, *C*_*max*_ Maximum plasma concentration, *CV* Coefficient of variation, *C*_*trough*_ Trough plasma concentration, *GeoMean* Geometric mean, *R*_*acc*_ Accumulation ratio, *t*_*1/2*_ Terminal elimination half-life, *T*_*max*_ Time to reach maximum plasma concentration, *V*_*z*_*/F* Apparent volume of distribution

After a single administration on day 1, SHR7280 was rapidly absorbed with a median time to reach the maximum plasma concentration (*T*_max_) ranging from 0.8 to 1.5 h across different dose groups. The *C*_max_ of SHR7280 in plasma and the mean AUC_0-12 h_ increased with the increasing doses of SHR7280, ranging from 696 to 6300 ng/mL and 1880 to 26,400 h*ng/mL, respectively.

On day 14, the median values of *T*_max_ in different dose groups of SHR7280 were generally consistent with those on day 1 (0.8–1.0 h); the exposure to SHR7280 (mean *C*_max_ and AUC_0-12 h_) increased in a dose-dependent manner. After reaching the peak, the concentration of SHR7280 declined, with similar mean *t*_1/2_ values among different dose groups (ranging from 2.8 to 3.4 h). The mean *CL*/*F* and *V*_z_/*F* values ranged from 29.2 to 59.7 L/h and 134 to 287 L, respectively. The mean *C*_trough_ ranged from 39.7 to 477 ng/mL, which was 5–8% of the mean *C*_max_ value in each group. Except for the 500 mg BID group, the mean *R*_acc_ calculated on the basis of AUC accumulation in the other dose groups was 1.1–2.7, indicating little to no accumulation of SHR7280 when the concentration of SHR7280 reached a steady state after multiple administration.

The correlation of *C*_max_ and AUC_0-12 h_ with the administrated dose was analyzed using an ANOVA model. Results showed that after dose normalization, both *C*_max_ and AUC_0-12 h_ did not have obvious intergroup differences, indicating a dose-proportional pattern of the PK parameters of SHR7280 (Additional file 1: Fig. S1).

### PD

All 70 participants who received either SHR7280 or placebo were included in the PD analysis.

**Fig. 2:**
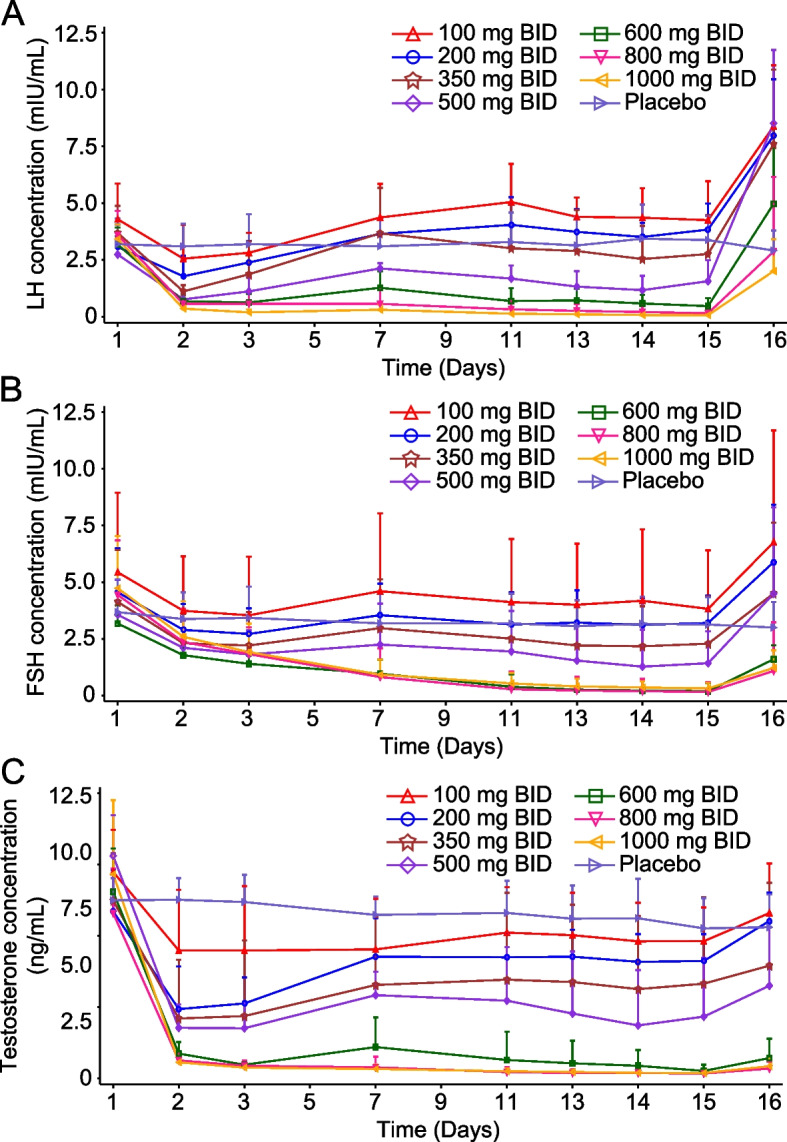
Effects of SHR7280 or placebo on LH (**A**), FSH (**B**), and testosterone (**C**) during 14 days of dosing in healthy men volunteers

The mean concentration of LH in healthy male subjects decreased rapidly after multiple administration (Fig. 2A). The inhibitory effect of SHR7280 on LH generally increased with increasing dose. Levels of LH in subjects receiving 500 mg or higher doses of SHR7280 generally remained lower than those of the placebo group after drug administration. SHR7280 doses of 800 and 1000 mg BID showed the strongest inhibitory effects on LH, and the mean LH levels in subjects from the two high dose groups remained below 1.0 mIU/mL from day 2 to day 14.

The FSH level of subjects from the SHR7280 groups gradually declined relative to placebo and then remained low after administration (Fig. 2B). The FSH level quickly rebounded after elimination of SHR7280. The inhibitory effects on FSH increased with increasing dose. The inhibitory effects of SHR7280 at doses of 600, 800, and 1000 mg BID on FSH were similar, and FSH levels became lower than 1.0 mIU/ml from day 7.

The testosterone levels in subjects who received SHR7280 rapidly declined relative to placebo following administration (Fig. 2C). A dose-dependent suppression of testosterone concentrations was observed in all SHR7280 groups, with the maximum testosterone suppression occurring in subjects receiving doses of 800 and 1000 mg BID. In these two high-dose groups, medical castration (testosterone concentration below 0.5 ng/mL) were achieved from day 2 and maintained even 48 h after the last dose. In other lower dose groups, partial testosterone suppression was obtained and rebounded between 24 and 48 h after the last dose.

### PK and PD correlations

Nonlinear regression fitting using the *E*_max_ model was performed to investigate the correlations between PK and PD parameters. The results showed that within the dose range of 100 to 1000 mg BID, decreased levels of LH, FSH, and testosterone were correlated with an increase in dose and AUC_0-t_ of SHR7280 (Additional file 1: Fig. S2). At doses of 600 mg BID and higher, a dose–response saturation trend in PD inhibition gradually became evident with increasing doses.

## Discussion

The GnRH antagonist SHR7280 demonstrated acceptable tolerability. PK results revealed a rapid onset of action and dose-dependent plasma exposure of SHR7280 within the dose range of 100 to 1000 mg BID, with minimal accumulation. PD results showed that SHR7280 inhibited LH, FSH, and testosterone concentrations in a dose-dependent manner. Medical castration was achieved at doses of 800 and 1000 mg from day 2 and maintained for 48 h after the last dose.

The safety results indicated good tolerability of SHR7280 in healthy men receiving SHR7280 tablets twice daily for 14 consecutive days within the dose range of 100 to 1000 mg. The incidence of AEs did not increase with increasing drug dose. The overall incidence of AE and treatment-related AE, as well as the severity of AE in SHR7280 group, were similar to those in the placebo group (incidence of AE, 76.8% vs 85.7%; incidence of TRAE, 75.0% vs 85.7%; incidence of moderate AEs, 1.8% vs 7.1%). Liver function-related AEs, such as increased blood bilirubin, increased alanine aminotransferase, and increased aspartate aminotransferase, occurred at similar incidence in the SHR7280 and placebo groups, were all mild in severity, and all resolved without special intervention. However, close monitoring of liver function indicators in participants receiving long-term use of SHR7280 is necessary in further studies due to the possibility that SHR7280 may have an impact on liver function. Additionally, the incidence of decreased blood calcium was higher in the SHR7280 group than in the placebo group. Although the severity was judged as mild, and the subjects did not experience fingertip numbness, tetany, or other symptoms, attention should be paid to decreased blood calcium in future studies of SHR7280. Common AEs, including hot flush and QTc interval prolongation observed with relugolix, were not reported in subjects treated with SHR7280 [17–19].

The PK profiles of SHR7280 were comparable to those of other oral GnRH antagonists, such as elagolix and relugolix [19, 20]. SHR7280 demonstrated a rapid onset of action, supporting its use as acute therapy. During twice-daily dosing of 100 to 1000 mg BID, no accumulation of SHR7280 was found. This finding was consistent with the PD data, which showed a rapid rebound of LH, FSH, and testosterone concentrations after elimination of SHR7280. In comparison with the peptide long-acting antagonist degarelix acetate for injection [21, 22], SHR7280, as a non-peptide small molecule antagonist, has certain advantages, such as high oral bioavailability, the ability to adjust the dose flexibly according to the weight and hormone levels of patients, and rapid clearance of the drug effect after discontinuation of administration.

The PD results of SHR7280 were also consistent with those reported for GnRH antagonists. SHR7280 demonstrated quick and sustainable suppression of LH, FSH, and testosterone levels, indicating its potential as a novel GnRH antagonist option. The prompt and rapid plasma exposure of SHR7280 may contribute to the quick response of sex hormones. The inhibitory effects on sex hormone were dose-proportional, and higher doses, such as 800, 1000, or even 600 mg BID, achieved medical castration of these hormones [23]. Relugolix, with a protocol-prespecified dose of 360 mg as an initial dose followed by daily doses of 120 mg, required 4 days to suppress testosterone level to achieve castration status in patients with advanced prostate cancer [17]; however, SHR7280 was able to suppress testosterone level to the castration status from day 2 in healthy male subjects, although the suppression effect by SHR7280 in patients with prostate cancer requires further validation. Taken together, these findings provide a basis for dose selection in the subsequent phase 2 and 3 clinical trials.

We found that the plasma concentration of SHR7280 was negatively correlated with declining levels of LH, FSH, and testosterone. Exploring the correlation between PK and PD parameters is helpful in guiding the optimal selection of drug dose and frequency in further clinical development since the association between PK and PD parameters represents the relationship between the optimal drug dose and clinical outcomes.

The study has several limitations. Firstly, the small sample size met the requirements for a dose-ascending phase 1 trial design but may have introduced bias into the final results. Secondly, efficacy data was not included as only healthy subjects were enrolled. Further evaluation of SHR7280 in larger cohorts of subjects is warranted. Thirdly, the administration period of the study drug was short. Considering that long-term drug castration therapy may cause severe psychological burden to healthy male subjects, we restricted the SHR7280 administration duration to 14 days. Fourthly, the healthy subjects enrolled in this study were younger than the typical age of prostate cancer patients. Elderly subjects are often prescribed more concomitant medications than younger patients, which may introduce bias in the interpretation of the safety, PK, and PD data of SHR7280. Therefore, we do not restrict enrollment to only elderly individuals. Meanwhile, we are conducting another clinical trial of SHR7280 (NCT04995042) to explore the safety, PK, PD, and efficacy of SHR7280 under a longer administration time (administration duration of 1 year) in patients with hormone-sensitive prostate cancer who are older than those in the current study.

## Conclusions

SHR7280 was well-tolerated for 14 consecutive days of use within a dose range of 100 to 1000 mg BID in healthy men. SHR7280 showed a rapid onset of action and was dose proportional. Its suppression of hormones was quick and reversible. These findings provide a rationale for further clinical development of SHR7280 as a potential androgen deprivation therapy for hormone-dependent diseases in men.

## Supplementary Information

**Additional file 1: Figure S1.** Dose-normalized PK parameters in different dose cohorts. **Figure S2.** Nonlinear regression fitting of E_max_ model demonstrated the correlations between PK and PD parameters.

## Data Availability

The datasets used and/or analyzed during the current study are available from the corresponding author on reasonable request.

## Abbreviations

ADT: Androgen deprivation therapy
AE: Adverse event
AUC_0-12_: Area under the plasma concentration–time profile
CL/F: Apparent total clearance
*C*_max_: Maximum plasma concentration
CTCAE: Common Terminology Criteria for Adverse Events
*C*_trough_: Trough plasma concentration
CYP3A: Cytochrome P450 3A
FSH: Follicle-stimulating hormone
GnRH: Gonadotropin-releasing hormone
LH: Luteinizing hormone
LLOQ: Lower limit of quantitation
MedDRA: Medical Dictionary for Regulatory Activities
PD: Pharmacodynamics
P-gp: P-glycoprotein
PK: Pharmacokinetics
*R*_acc_: Accumulation ratio
RTSM: Randomization and Trial Supply Management
SRC: Safety Review Committee
*t*_1/2_: Half-life
*T*_max_: Time to maximum plasma concentration
*V*_z_/*F*: Apparent volume of distribution
