# Supplementary material for: Safety, pharmacokinetics, and pharmacodynamics of SHR7280, an oral gonadotropin-releasing hormone receptor antagonist, in healthy men: a randomized, double-blind, placebo-controlled phase 1 study
Source: BMC Med. 2023 Apr 3;21:129. doi: 10.1186/s12916-023-02834-6 (PMC10071678; doi:10.1186/s12916-023-02834-6)
Supplement: Supplementary file 1 — Additional file 1: Figure S1. Dose-normalized PK parameters in different dose cohorts. Figure S2. Nonlinear regression fitting of Emax model demonstrated the correlations between PK and PD parameters. [file 12916_2023_2834_MOESM1_ESM.pdf]

Figure S1. Dose-normalized PK parameters in different dose cohorts.

(A) Box-whisker plot of dose-normalized C_{max} of SHR7280; (B) Box-whisker plot of dose-normalized AUC_{0-12} of SHR7280.

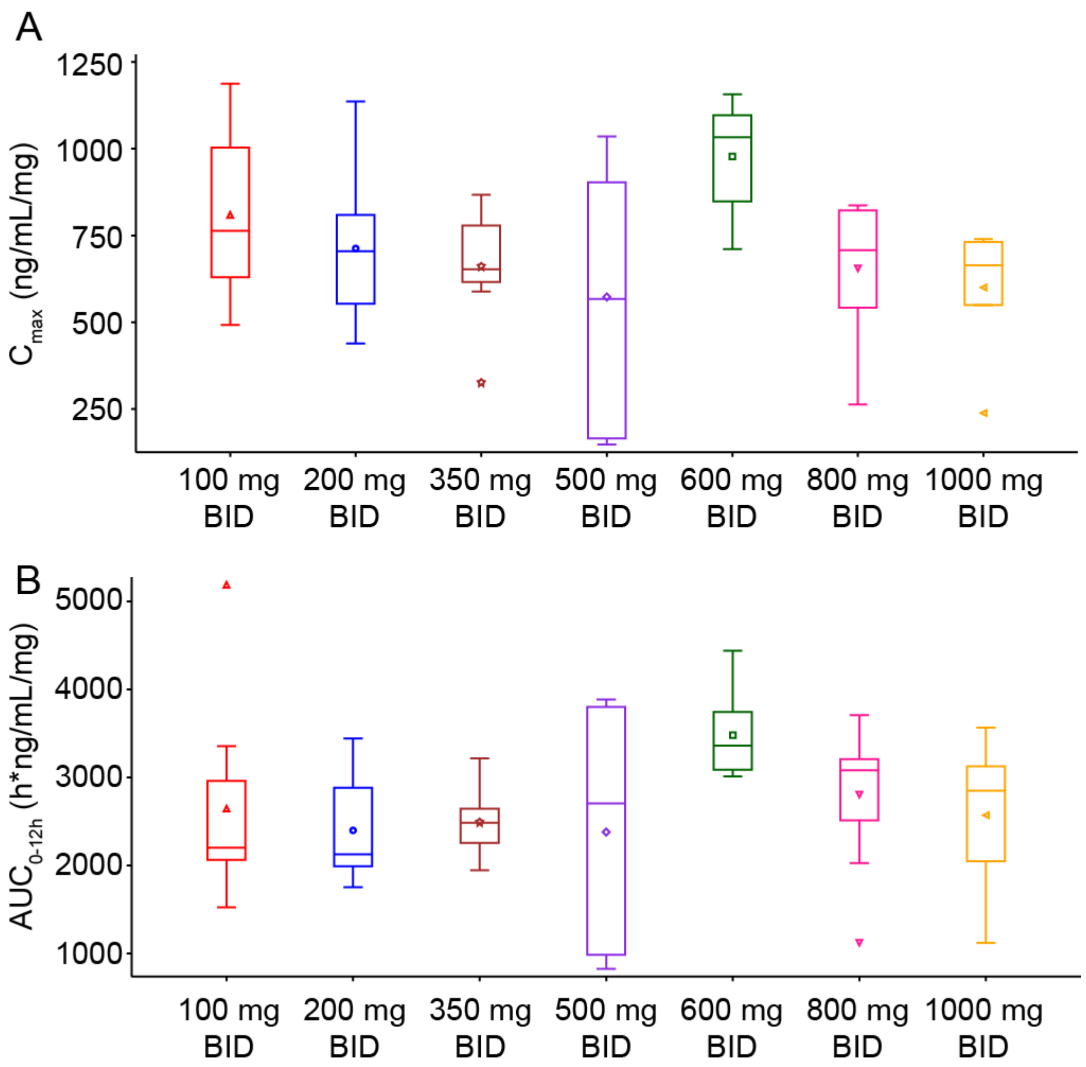

Figure S2. Nonlinear regression fitting of E_{max} model demonstrated the correlations between PK and PD parameters.

(A) Correlation of LH AUC_{0-14d} with SHR7280 dose. (B) Correlation of LH AUC_{0-14d} with SHR7280 AUC_{0-12h}. (C) Correlation of FSH AUC_{0-14d} with SHR7280 dose. (D) Correlation of FSH AUC_{0-14d} with SHR7280 AUC_{0-12h}. (E) Correlation of T AUC_{0-14d} with SHR7280 dose. (F) Correlation of T AUC_{0-14d} with SHR7280 AUC_{0-12h}.

A

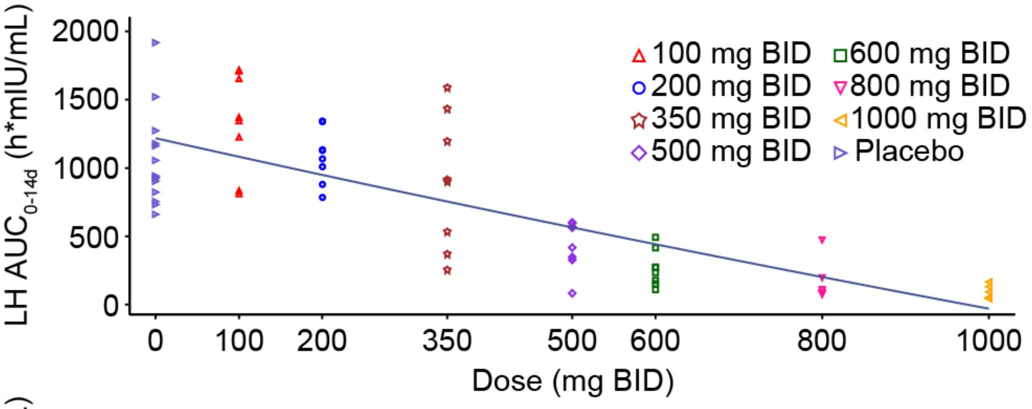

B

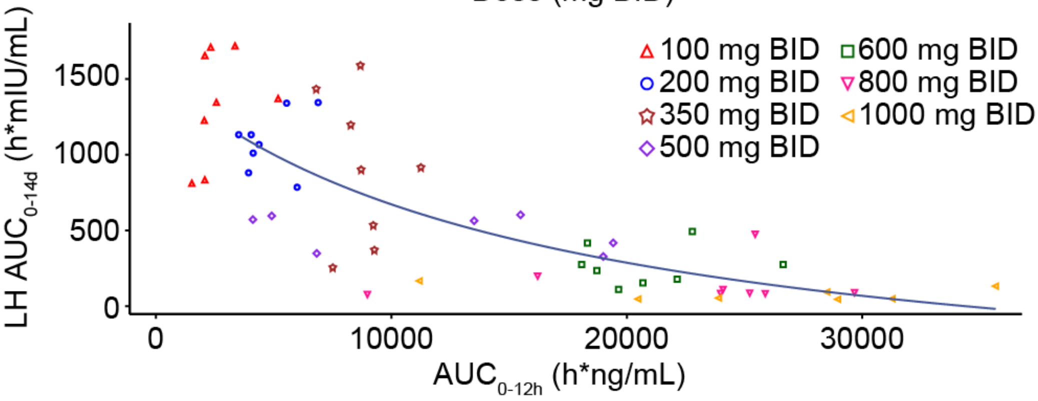

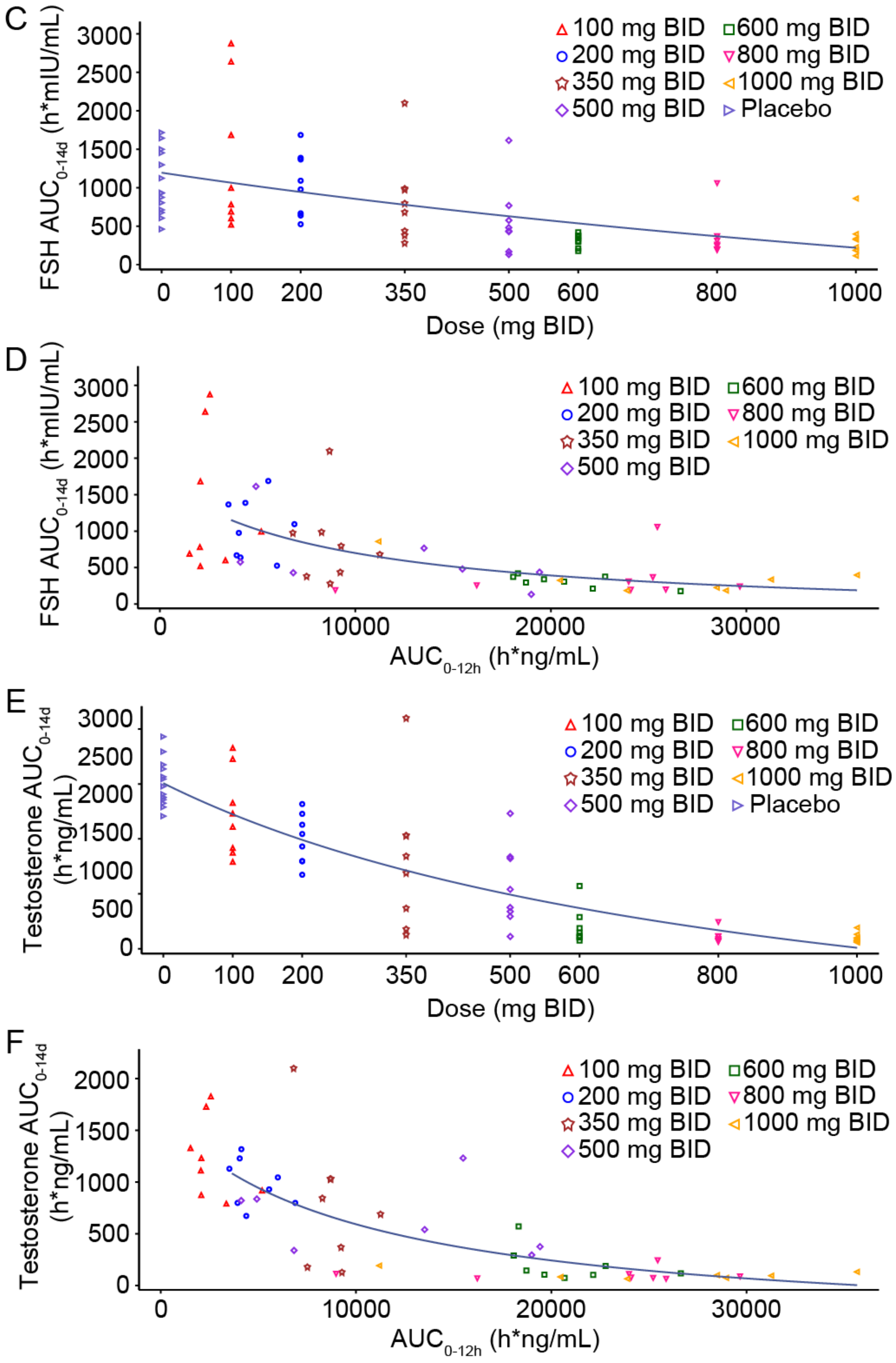
C
FSH AUC0-14d (h*mIU/mL)
Dose (mg BID)
100 mg BID
200 mg BID
350 mg BID
500 mg BID
600 mg BID
800 mg BID
1000 mg BID
Placebo
D
FSH AUC0-14d (h*mIU/mL)
AUC0-12h (h*ng/mL)
E
Testosterone AUC0-14d (h*ng/mL)
Dose (mg BID)
F
Testosterone AUC0-14d (h*ng/mL)
AUC0-12h (h*ng/mL)
